# Keystone Veterinary Medical Association

**Published:** 1892-07

**Authors:** W. S. Kooker

**Affiliations:** Secretary


### SOCIETY PROCEEDINGS.

---

*Keystone Veterinary Medical Association.*—The regular monthly meeting of the Keystone Veterinary Medical Association was held in the hall of the College of Physicians, Philadelphia, on Tuesday evening, May 9th, at 8 P. M. Dr. Hoskins, President, called the meeting to order, and on roll call, members Hoskins, Kooker, Luitz, Weber, Webster, Sellers answered to their names Drs. W. L. Nunan and J. T. Ferley, Jr., were present and made application for membership. Their names were referred to the board of Trustees.

The essayist for the evening, Dr. Chas. Luitz, read a paper entitled "Laminitis," covering the ground with special reference to treatment. It was followed by an interesting discussion, showing many forms of treatment and all having something to commend them. The relative value of heat and cold was a source of interest and a difference of opinion.

Dr. Hoskins reported a case of "Parotid Fistula," which had proven rebellious to treatment covering a period of six months and which, there was reasons to believe, had been in process of development for some six months previous to opening. It finally discharged a piece of woody fibre, which resembled the body of a wheat bead, after which it healed up rapidly.

Dr. S. E. Weber gave a brief outline of the scope of his researches in the diseases of Rodents, and spoke of the unusual prevalence of tubercular lesions. The President requested him to prepare an article upon the subject for the June meeting after which the meeting adjourned.

W. S. KOOKER, *Secretary.*

---

*Keystone Veterinary Medical Association.*—The regular meeting of the Keystone Veterinary Medical Association was held at the College of Physicians, June 14th, 1892; the President, W. Horace Hoskins in the chair. The minutes of May meeting were read and approved. Bills for hall rent, printing and postage were ordered paid. There being an absentee in the Board of Censors, the President appointed Dr. Bridge to act in his stead. They reported favorably for membership Drs. J. T. Ferley and Wm. L, Nunan. The report was accepted, and the gentlemen duly elected. The President announced the death of the Hon. John S. McKinley a noted member of the bar, as well as a friend of the Veterinarian, and the author of a recent paper before the Association on the law of warranty; he called upon Dr. Kooker for remarks. The speaker paid a tribute to the jurist, as a scholar, worker, and a fast friend. There were remarks by others present, when the President appointed Drs. Kooker and Weber a committee to draw up suitable resolutions to be presented to the family. The President paid a glowing tribute to the memory of Dr. Henri Formad, a member of the Association, and was followed by Drs Bridge, Weber, and Kooker. Drs. Huidekoper, Bridge and Kooker were appointed a committee to draw up resolutions expressive of the sentiments of the Association.

Dr. S. E. Weber read a paper upon rats, as a scourge of the human being and a propagator of disease.

W. S. KOOKER, *Secretary.*

